# The validity of self-reported drug use with urine test: results from the pilot phase of Azar cohort study

**DOI:** 10.15171/hpp.2018.30

**Published:** 2018-07-07

**Authors:** Shahnaz Ashrafi, Nayyereh Aminisani, Somaieh Soltani, Parvin Sarbakhsh, Seyed Morteza Shamshirgaran, Mohammad-Reza Rashidi

**Affiliations:** ^1^Epidemiology and Statistics Departement, Tabriz University of Medical Sciences, Tabriz, Iran; ^2^Department of Medicinal Chemistry, Tabriz University of Medical Sciences, Tabriz, Iran

**Keywords:** Validation, Self-report, Prevalence, Substance abuse, Drug, PERSIAN cohort

## Abstract

**Background: ** The present study aimed at assessing the validity of self-reported drug use in people aged 35 and older in a pilot phase of a population-based cohort study.

**Methods:** A total of 1038 adults over 35 years old in Khamene city in East Azerbaijan province were recruited for the pilot phase of Azar cohort; a province-level of a nationwide PERSIAN cohort study completing a questionnaire and providing biological samples from October to December 2014. Information about the history and duration of smoking tobacco, using drug and medication were obtained by the physician. The validity of the drug use was assessed through comparing the questionnaire response with three urine strip tests for the detection of morphine, amphetamine and methamphetamine among 259 randomly selected subjects.

**Results: ** The prevalence of drug use according to self-report was 2.6% (95% CI: 1.7%-3.8%).One-step drug test as the gold standard for the use of drug self-reported demonstrated a sensitivity(95% CI) and specificity 15% (10-22) and 99.7% (98.9%-99.9%) respectively. All participants with positive self-report were male; however, in the urine analysis drug test, it was positive for 7out of 68 randomly selected women.

**Conclusion: ** The validity of self-reported drug use in this population was low; therefore, the self reported use of the drug should be used with caution in this population. It is recommended to use alternative techniques to improve the validity of data using the self-report procedure.

## Introduction

In 2015, it was estimated that 250 million people or 5% of adults around the world had used an illicit drug at least once during their life.^1^ In 2015, reports showed that illicit drug dependence was directly responsible for 27.8 million disability adjusted life years (DALYs: 95% uncertainty interval [UI] 24.4-31.2 million) accounting for about 1% of global all-cause DALYs. Globally, the dependence on opioids and amphetamines is more common than dependence on other drugs. Opioid dependence is accounted for the largest burden of DALYs (12 million), throughout the world.^2^

Drug use is measured in a clinical setting, workplace, the justice system, and population survey for different reasons. In epidemiologic studies, the prevalence of drug use, its pattern and correlations are often measured using the self-report method.^3^ However, because the drug use is categorized as sensitive and highly stigmatised behaviours, the validity of self-reported data has been questioned. Despite recent advances in bioassay and methodology contributing to improve drug use estimates, researchers continue to rely on the self-reported drug use in epidemiological studies because of the time, cost and resource considerations. The most important biological marker-based validity information comes from criminal justice ^4^ and treatment setting.^5^ There are studies assessing validation of self-reported drug use with biochemical testing among patients presented to the emergency department seeking treatment for backache, headache, and toothache, however, population-based validity studies are scarce.^6^

Due to its specific socio-cultural beliefs and geographical situation, Iran has a major public health problem regarding drug and alcohol use. It accounted for 804.5 DALYs in men and 227 in women.^7^ History of opium use as medicinal and recreational substance in Iran dates back to more than four centuries.^8^ However, estimating a definite prevalence of illicit drug use in Iran is not possible due to high stigmatisation and legal restriction. Recent results of a national household survey in Iran reported that the prevalence of illicit drugs, among which opium was the most common type, was 2%.^9^ Iran has shown the highest rate of opiate abuse in the world. In recent years, there has been the increased use of heroin, crystal methamphetamine, and ecstasy.^10^

Drug use as an adverse health behaviour linked to morbidity and mortality has been considered in cohort studies as well.^11^ However, in these studies similar to cross-sectional surveys,^12^ data is oftenmeasured using self-report administration. Therefore, the validity of self-reported drug use in cohort studies is important. Abnet et al^13^ reported a high validity of self-reported opium use in a population at high risk for oesophagal cancer in northern Iran (sensitivity = 93%, specificity =89%). However, it must be considered that opium is widely used in that population^13^ and there is no social stigma for drug self-reporting in that region but in other provinces, and the results for other regions may not be as reliable as Abnet and colleagues’ report in northen Iran.^13^ Anecdotal evidence suggests that drug use among Azari people in the northwest of Iran is not socially accepted, while Turkmen in north of Iran use opium as traditional medicine with no social stigma.^13^

Sociocultural factors can shape people’s self-reported drug use, consumption patterns and legal restrictions on substance misuse, then similar studies like Abnet and colleagues’ research in northern Iran, are necessary. This study aimed at examining the prevalence and validity of self-reported drug use in the pilot phase of Azar cohort study; a province-level of the Prospective Epidemiological Research Studies of the Iranian adults (PERSIAN) which is a nationwide cohort study launched in 2014.^14^

## Materials and Methods

### Study subjects and recruitment 

The Azar cohort is a province-level of the PERSIAN cohort study which mainly aimed at assessing a comprehensive range of different biomarkers, clinical, lifestyle, and socioeconomic factors of common non-communicable diseases among Iranian adults. The present study was based on the data from the pilot phase of the Azar cohort study which was conducted in Khamene city in East Azarbaijan province. All individuals 35 years and older who agreed to participate in the study were included in the current analysis. A total of 1236 individual were invited among whom, a total of 1038 subjects participated in the study (participation rate 84%). The participants were invited to visit the Azar cohort centre located in Khamene city. Trained interviewers completed a comprehensive questionnaire, and biological samples (urine, blood, hair and nail) were collected from October to December 2014.

### Questionnaire and sample collection 

As part of the data collection procedure for the PERSIAN cohort, an interviewer-administered electronic questionnaire consisting of 55 questions and 482 items was developed, encompassing many different aspects of an individual’s life that may influence their health status.^14^ One of the major categories of Azar cohort’s questionnaire was about using tobacco and drugs. Other variables in this questionnaire include sex, age, occupation, marital status, education level, disease and medicine use, physical activity, etc. Due to budget consideration, we had to select up to 300 hundred cases for the laboratory tests. Therefore, for the purpose of this study, all individuals who had positive self-report (n = 27) and a random sample of 259 individuals were selected from 1011 participants who had negative self-report, via generating random numbers in Excel. According to the previous studies in Iran,^12,15^ drug use is more frequent among men than women, so the number of randomly selected cases of men was higher than that of women. In the validation study, 68 women and 191 men were randomly selected. Few cases (n = 15) were tested twice as the process of validation.

### Laboratory analysis

At the time of data collection, 4 mL urine sample was collected from all subjects (1038 samples) and got frozen. They were kept at -20°C refrigerator in Azar cohort centre. Selected samples were transferred from cohort centre to central laboratory, Pharmacy Faculty in Tabriz University of Medical Sciences using insulation boxes containing dry ice. The samples were kept at -70°C until analysis. Urine samples were tested for morphine, amphetamine and methamphetamine using the one-step AMP (amphetamine), MET (methamphetamine) and MOP (morphine) Rapid Urine Test Strip (Wondfo, China). The detection is based on lateral flow, one-step immunoassay for the qualitative detection of specific drugs and their metabolites in human urine. The results should be read and evaluated within 5 minutes. Applied one step urine tests has set the screen cut-off for positive specimens at 500 ng/mL for d-methamphetamine and d-amphetamine and 300 ng/mL for Morphine in urine at 5 minutes.^16^ These urine strips were selected based on the previous studies about the common drugs of abuse in the country.^17^ Many Amphetamine drugs have their street names as follows: uppers, bennies, black beauties, speed, eye-openers, lid poppers, pep pills, wake-ups, and white crosses. Street name for Methamphetamine: crack, crystal, crystal meth, meth, go-fast, speed, zip, heroin and speedballs.^18^ The most notorious derivative of morphine is heroin^19^ and opium.

All positive samples were checked for the complexity of interfering substances, for example, Acetoacetic Acid, Acetone, Albumin etc by a pharmacist according to participants’ response to the questionnaire.

To do the urine analysis, the daily studied samples were transferred into the room temperature 30 minutes before the analysis and allowed to thaw at room temperature. An equal volume of each sample transferred into 2 mL microtubes, and the absorbent end of the test strips was immersed in the urine sample. A random sample was retested daily for checking the reliability of the strips. Twelve samples were tested twice, and similar results were obtained for all.

Results were recorded as positive or negative based on the manufacturer’s cut-off values: Morphine (300 ng/mL), Amphetamine and Methamphetamine (500 ng/mL). A final assessment was announced as positive result base on the presence of one of these substances alone or in combination with other substances.

### Data analysis

Data were presented using, mean, standard deviation (SD) for quantitative variables and frequency for qualitative variables. We assessed the validity of self-reported drug use by comparing the results of the questionnaire to the results of the urine strips in the subgroup of 284 participants with both measurements. We calculated the sensitivity and specificity using two approaches; un-weighted and weighted. All participants with positive self-report (n = 25) and a random sample of participants with negative self-report (n = 259) were tested using the one-step rapid urine test strips (unweighted approach). Urine sample was not available for two positive cases. Due to the dipropionate stratification of laboratory tested sample, we used the weighted approach. We assigned an adjustment weight based on the results of the random sample of negative self-report cases to all study participants to calculate the sensitivity and specificity. Besides, the results for men were also reported separately because the self-report of drug use is so different for men and women in Iran. We also performed Bootstrap method to estimate the confidence interval due to low sample size, and the results were similar to the weighted routine CI. All analyses were performed using STATA (version 14), the statistical software.

## Results

A total of 1038 participant aged 35 and older were included in the pilot phase of the Azar cohort study. The mean age was 52, SD = 11.7 years, 44.8% of them were: male, 88% married and currently living with their spouse, and 23.8% current smokers. The majority of women were housewives (74%), 21% of the study participants were workers, and less than 10% were illiterate (Table 1).

The prevalence of drug use was 2.6% (95% CI: 1.7%-3.8%), based on self-report; 27 subjects had positive self-report of drug use. All participants with positive self-report were male, in age from 42 to 69 years old (Mean = 54.8, SD = 7.3). For validation, we selected a random sample of 259 individuals who were negative self-reported drug user plus those who had reported that they were a drug user. We compared the results of urine drug test of 284 individuals with the results of their self-report. The urine results were not available for 2 positive self-report drug users. Drug use was positive in 54 cases (19.01%) of the sample. Those who had reported that they had not used the drug in the past 48 hours, 32 subjects (25 men and 7 women) tested positive for the drug on the urine test. Among those who were self-reported drug users, the urine test was positive for 22 out of 25. The general characteristics of those with negative self-report but positive urinalysis (32 subjects) were similar to others except they were more likely to be men (*P* < 0.001) and smoker (*P* = 0.003).

Of all participants, 19% of the samples were positive in urinalysis, 2.6% (95% CI: 1.7%-3.8%) of whom reported drug use, 81% tested negative and reported no drug use, and therefore, concordance rate was 87.7% (249 out of 284) between self-report and urine test data.

Among 54 participants with a positive urine test, 30 individuals were found to have morphine in their urine, 22 tested positive for methamphetamine, and four positive samples for amphetamine.

The weighted sensitivity (95% CI) and specificity (95% CI) of self-reported drug use was 15% (10%-22%) and 99.7% (98.9%-99.9%), respectively (Table 2). Then, we stratified the analysis according to gender because drug use was not reported by any women. We found the weighted sensitivity of 24.7% (16.5%-35.2%) and the specificity of 98.2% (99.2%-97.5%) for men (Table 3). The sensitivity and specificity were lower based on the unweighted approach because we included all positive self-reports and a relatively small random sample of negative self-reports (data were not shown). In addition, the positive likelihood rate (LR+) was 44.35 (13.44–146.31), the negative likelihood ratio (LR-) was 0.85 (0.80–0.91), the positive predictive value (PPV+) was 88.0% (69.0%-96.0%), and the negative predictive value (PPV-) was 87.6% (86.9%-88.5%).

## Discussion

The current study aimed at assessing the validity of the self-reported drug use in the pilot phase of the Azar cohort study, in the northwest of Iran. The prevalence of self-reported drug use in this study sample was 3% which was lower than a study conducted by Abnet et al in the north of Iran with 8.8%.^13^

We found a low sensitivity and high specificity of self-reported drugs use with urine test analysis as gold standard. Our finding confirms the popular belief that nonusers often tell the truth, while some users deny it is indicating the high specificity and low sensitivity. However, the sensitivity was lower in our study compared to other studies which reported a sensitivity of 93%. The reason for this might be explained partially by the socio-cultural beliefs and ethnicity differences between two areas. The Azar cohort was conducted among Azari people in the northwest of Iran where the drug use is not socially accepted, while the study by Abnet et al^13^ was conducted among Turkmen in northern of Iran where opium is used as the traditional medicine with no social stigma.

In comparison to biological markers, self-report data are usually cheaper to obtain, more practical and allow researchers to gather more detailed information about longer-term drug use behaviours, routes of use, and context of drug-use behaviours. Although the collection of self-report data is often the only viable approach toward assessing the drug use, responses may be influenced by factors such as presentation biases, social desirability, or a participant’s ability to recall information. Social stigma associated with drug use or fear of legal repercussions may lead participants to be unwilling to report having used this illicit drug. Alternatively, people may exaggerate their drug use to meet eligibility requirements for a program, be seen as rebellious, or impress an interviewer,^20^ but for this population, self-reported is not a valid measure to evaluate the prevalence of drug use.

Self-reported drug use among women was 0% in the questionnaire, but in urinalysis, we found that about 22% of those with negative self-reported drug use were female. Therefore, in planning to achieve the status of drug users and future programs, it must be noted that self-reported in this population cannot be reliable, especially for women.

This study has some strengths and limitations. It highlights that the self-report cannot be solely used as the tool for evaluation of substance use in a population-based study. The main limitation of the current study was the inability of doing the urine test for all participants due to limited resources. Therefore, the urine test was performed for all positive self-reported cases and in sub-sample of the study participants who were selected randomly. However, we applied weighting adjustment for disproportionate stratification to overcome this problem partially. Secondly, the results in men and women were not comparable because of low false+ specially in women; there is a need to further research among women with sufficient sample size. Another limitation might be using a urine analysis as gold standard while the other biological markers like hair could be better markers to identify the drug’s metabolites over a longer period. However, at the time of this study, the protocol for using other biological samples like hair was not available. Also, a urine test is considered as a simple, rapid, and less expensive measure for assessment of self-reported drug use in epidemiological studies.

## Conclusion

In conclusion, according to the findings mentioned above, it is recommended to use both self-report and biological measures to provide a better estimation of this population. Special attention should be given to females that might deny it more than men due to sociocultural issues. Further research using other biological markers are also needed.

## Ethical approval

This study was approved by the Ethics Committee in Tabriz University of Medical Sciences (TBZMED.REC.1394.1201). Signed informed consent was obtained from all participants of the AZAR cohort study before the data collection.

## Competing interests

Authors declare no conflict of interest.

## Authors’ contributions

NA, MR and SMS contributed in original idea and protocol, the conception of the work, conducting the study, revising the draft, approval of the final version of the manuscript, and agreed for all aspects of the work. PS and SS contributed in the design of the work; SS supervised the laboratory tests, PS performed the analysis. PS and SS revised the draft and approved the final version of the manuscript. SA contributed to the conception of the work, conducting the study, wrote and editing of this manuscript.

## Acknowledgments

This research was conducted under a thesis grant for the Master’s degree from the Department of Statistics & Epidemiology, Tabriz University of Medical Sciences. We gratefully acknowledge the AZAR cohort Study team for their collaboration during the research process.

**Table 1:** General characteristics of stud participants in the pilot phase of the Aar cohort stud 2014

	**No.**	**%**
Age group		
<45	273	26.4
45-54	390	37.7
55-64	216	20. 9
65+	155	15.0
Sex		
Male	465	45.0
Female	569	55.0
Marital status		
Married	912	88.1
Single/divorced/widow	123	11.9
Education		
Illiterate& elementray	399	38.6
Secondary & high school	373	36.1
University	262	25.3
Occupation		
Unemployed/ retired	104	10.3
Worker	217	21.4
Self- employed	133	13.1
Clerk	139	13.7
Housewife	420	31.5
Smoking		
No	789	76.2
Yes	247	23.8

* Total numbers is not the same due to missing values.

**Table 2:** Validity of self-reported drug use comparing questionnaire responses to urinalysis (weighting adjustment)

	**Urine analysis**		
	**Yes**	**No**	**Total**
Self-reported drug use			
Yes	22	3	25
No	125	886	1011
Total	147	889	1038

Sensitivity (95% CI): 15% (10%-22%). Specificity (95% CI): 99.7% (98.9%-99.9%). Positive Likelihood Rate (LR+): 44.35 (13.44–146.31). Negative Likelihood Ratio (LR-): 0.85 (0.80–0.91). Positive Predictive Value (PPV+): 88.0% (69.0%-96.0%). Negative Predictive Value (PPV-): 87.6% (86.9%-88.5%).

**Table 3:** Validity of self-reported drug use comparing questionnaire responses to urinalysis in men (weighting adjustment)

	**Urine analysis**		
	**Yes**	**No**	**Total**
Self-reported drug use			
Yes	22	3	25
No	67	373	440
Total	89	376	465

Sensitivity (95% CI): 24.7% (16.5%-35.2%) for men. Specificity (95% CI): 98.2% (99.2%-97.5%) for men.
